# 2D Rhenium- and Niobium-Doped WSe_2_ Photoactive Cathodes in Photo-Enhanced Hybrid Zn-Ion Capacitors

**DOI:** 10.1021/acsanm.4c01405

**Published:** 2024-06-18

**Authors:** Monaam Benali, Jalal Azadmanjiri, Martin Loula, Zhongquan Liao, Rui Gusmão, Amutha Subramani, Kalyan Jyoti Sarkar, Rabah Boukherroub, Zdeněk Sofer

**Affiliations:** † Department of Inorganic Chemistry, University of Chemical and Technology-Prague, Technicka 5, 166 28 Prague 6, Czech Republic; ‡ Institute of Organic Chemistry and Biochemistry, Czech Academy of Sciences, Flemingovo nám. 2, 166 10 Prague 6, Czech Republic; § Fraunhofer Institute for Ceramic Technologies and Systems IKTS, Maria-Reiche-Straße 2, 01109 Dresden, Germany; ∥ Univ. Lille, CNRS, Univ. Polytechnique Hauts-de-France, UMR 8520, IEMN, F-59000 Lille, France

**Keywords:** TMDs, doping, photoconversion, energy storage, Zn-ion capacitor

## Abstract

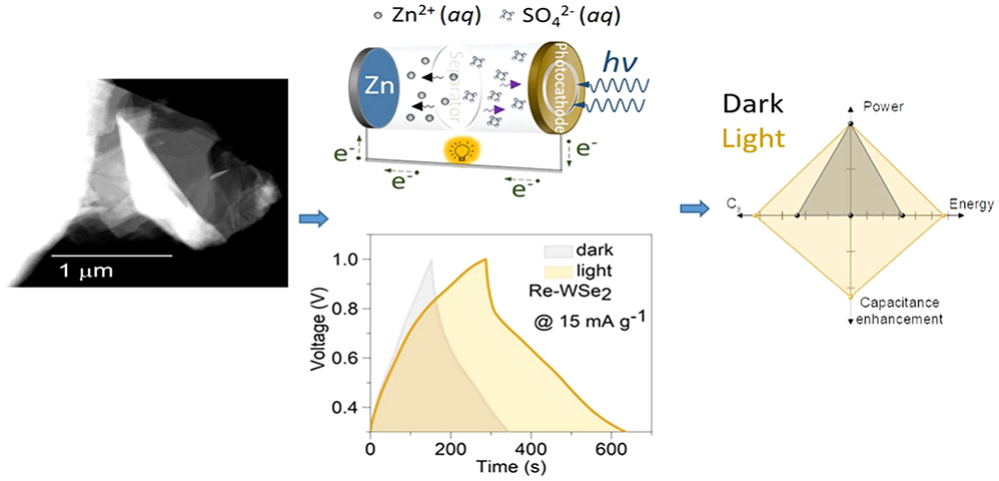

Designing a multifunctional device that combines solar energy conversion and energy storage is an appealing and promising approach for the next generation of green power and sustainable society. In this work, we fabricated a single-piece device incorporating undoped WSe_2_, Re- or Nb-doped WSe_2_ photocathode, and zinc foil anode system enabling a light-assisted rechargeable aqueous zinc metal cell. Comparison of structural, optical, and photoelectric characteristics of undoped and doped WSe_2_ has further confirmed that ionic insertion of donor metal (rhenium and niobium) plays an important role in enhancing photoelectrochemical energy storage properties. The electrochemical energy storage cell consisting of Re-doped WSe_2_ (as the photoactive cathode and zinc metal as anode) showed the best photodriven enhancement in the specific capacitance of around 45% due to efficient harvesting of visible light irradiation. The assembled device exhibited a loss of 20% of its initial specific capacitance after 1500 galvanostatic charge–discharge cycles at 50 mA g^–1^. The cell also provided a specific energy density of 574.21 mWh kg^1–^ and a power density of 5906 mW kg^1–^ at 15 mA g^–1^. Under otherwise similar conditions, the pristine WSe_2_ and Nb-doped WSe_2_ showed photoenhanced induced capacitance of 43% and 27% at 15 mA g^–1^ and supplied an energy density of 436.4 mWh kg^1–^ and 202 mWh kg^1–^, respectively. As a result, a reasonable capacitance improvement obtained by the Re-WSe_2_ photoenhanced zinc-ion capacitor could provide a facile and constructive way to achieve a highly efficient and low-cost solar-electrochemical capacitor system.

## 1 Introduction

Solar-driven self-powered systems have become crucial for the sustainability of consumer electronics, electric vehicles, and off-grid storage devices.^1,2^ Advanced photoelectrochemical energy harvesting and storage technologies are part of this dynamic and have proven high potential in the conversion and storage of solar energy.^3,4^ Recently, multivalent ion storage devices, including zinc (Zn) and magnesium (Mg) elements, have gained considerable attention due to their high energy density, natural abundance, ecofriendliness, and cost-effectiveness. Particularly, multivalent Zn-ion batteries and supercapacitors (SCs) exhibit some distinct advantages of greater cycling stability, intrinsically high safety, and reliability compared to other univalent metal (K^+^, Li^+^, Na^+^, etc.) energy storage systems.^5,6^ Scientists are currently working to improve and optimize various electrode materials, electrolytes, and cell designs to enhance the overall performance of Zn-ion devices. Moreover, Zn displays limited dendrite formation during the plating and stripping processes. Researchers aim to improve the energy density, power output, and lifespan of Zn-ion supercapacitors and batteries by investigating the fundamental aspects related to the Zn-ion intercalation, charge storage mechanisms, and ion transport kinetics. These efforts may pave new avenues for the conception of more sustainable and efficient energy storage solutions.

Recently, photoenhanced Zn-ion capacitors (photo-ZICs), in which a photoelectric conversion (PC) unit is integrated with a rechargeable electric energy storage system (EESS) in one device, have been explored owing to their potential for fast light harvesting and energy storage.^7,8^ For instance, incorporation of porous carbon together with cadmium sulfide-enrobed zinc oxide nanorods (PC/CdS@ZnO)^9^ and molybdenum disulfide-zinc oxide (MoS_2_–ZnO)^10^ has been considered in different photoassisted energy storage systems. The attention of scientists worldwide has been focused on using simultaneous photoactive and capacitive (or pseudocapacitive) materials for harvesting and storing inexhaustible solar energy. In this way, a semiconductor absorbs light and provides separated electron–hole pairs. The electrons transverse the external circuit toward the anode side. The holes accumulated on the electrode/electrolyte interface are captured by the anions existing in the electrolyte (holes will interact with SO_4_^2–^ ions from the ZnSO_4_ electrolyte in the case of photo-ZIC). The accumulated electrons on the Zn anode will attract the cations of the aqueous electrolyte. Then, the positive charge in the electrolyte are stored in the electric double layer on the top surface of the bifunctional material.^11^

Among different types of nanomaterials, 2D nanostructures possess high surface area, edge sites, and tunable band gap. These characteristics make 2D nanomaterials effective in electrochemical capacitors. For instance, 2D transition-metal dichalcogenides (TMDCs, semiconductors type of MX_2_, where M is a transition metal including W and Mo, and X stands for chalcogen S, Se, or Te), hold tremendous potential for the development of efficient and sustainable technologies to address growing energy demands. Exploring light-matter interactions has become a prominent focus in various fields, due to the recognition that light can significantly enhance the performance of electrochemical energy storage systems. This intensified interest arises from the ability of light to either fully charge these systems or improve their charging rates and capacitance. Combining energy harvesting and -storing capabilities in a single structure reduces fabrication costs and package volume. Additionally, advancements in fabricating a single-layer WSe_2_ or TMDCs, which typically displays a direct narrow band gap, can enhance the light-harvesting ability of these materials. Consequently, the development of dual-functional structures that rely on a single electrode for both energy harvesting and storage has the potential to improve the efficiency of WSe_2_-based energy storage and reduce energy loss during transmission compared to other photoelectrode-based zinc ion capacitors.

**Scheme 1:**
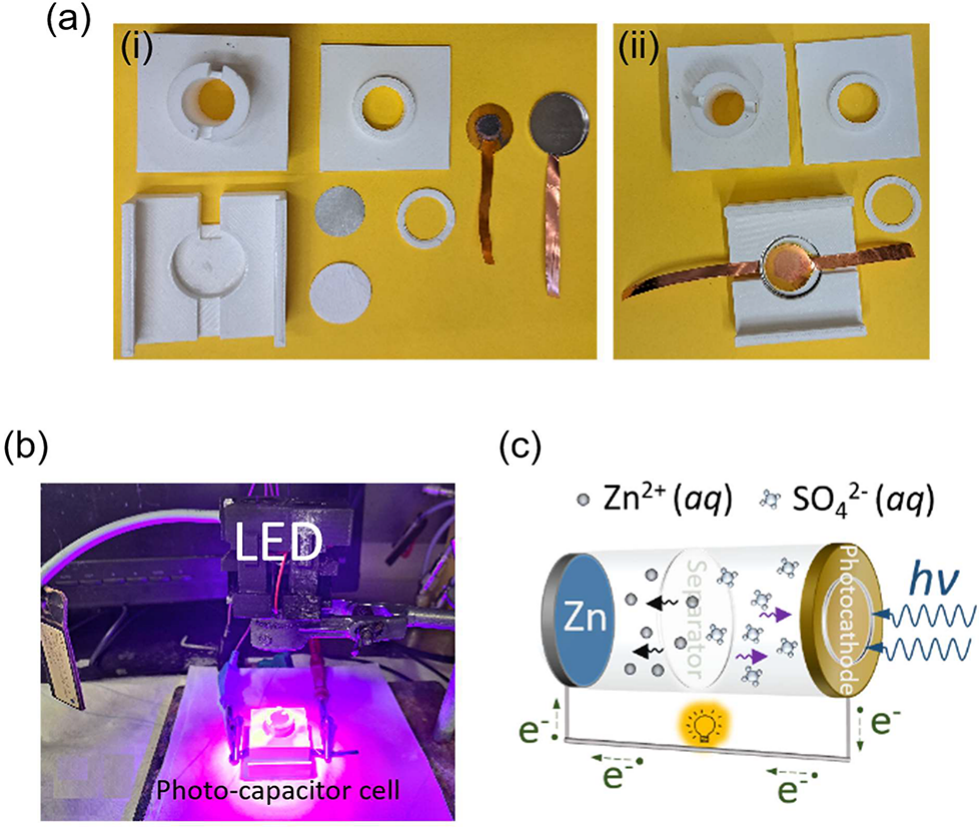
Experimental Setup of Photo-ZIC Device (a) A coin cell type 2016 was placed inside a printer holder allowing an optical window with a diameter of 8 mm for light irradiation. 100 μL of ZnSO_4_·7H_2_O aqueous solution was dropped to ensure wetting of the separator. The connector was crafted using polylactic filament PLA from Prusa polymer. A printed threaded rod with a hole in its center was used to seal the holder and protect the cell and electrolyte. (b) ZIC device under LED. (c) Mechanism of photo-ZIC device.

Hence, this study reports the first photoenhanced ZIC using Re- or Nb-doped WSe_2_ as a photoactive material. We demonstrate that light could enhance the capacitance and rate performance of photoenhanced ZICs. Doped WSe_2_ coated on transparent substrate was paired with a zinc foil as an anode electrode (Scheme 1) to create photoenhanced Zn-ion capacitor devices. The asymmetric electrode design studied in the present work allows light absorption and a capacity of charge storage.

## 2 Experimental Details

### 2.1 Materials

Zinc sulfate heptahydrate (ZnSO_4_·7H_2_O) and Zn metal foil were purchased from Sigma-Aldrich. Tungsten powder (99.999%, ∼100 mesh, China Rhenium Co., China), niobium powder (99.9%, Beijing Metallurgy and Materials Technology Co., China), rhenium powder (99.999%, ∼100 mesh, China Rhenium Co., China), selenium granules (99.9999%, 2–6 mm granules, Wuhan Xinrong New Material Co., China), and selenium bromide (99.9%, Strem, USA) were used as-received. Poly(vinylidene fluoride) (PVDF, 99%) binder for the ZIC cell was obtained from Alfa Aesar. Carbon black (super P, 99%) and *N*-methylpyrrolidone (NMP, 99%) were procured, respectively, from Acros Organics and Thermo Fisher Scientific.

### 2.2 Synthesis of Pristine WSe_2_, Rhenium- and Niobium-Doped WSe_2_

The undoped and doped tungsten diselenide (WSe_2_) were prepared by direct reaction from elements in a quartz ampule with subsequent chemical vapor transport. For the synthesis of WSe_2_ crystal, 50 g of tungsten and selenium were placed in an ampule (50 × 250 mm) together with 1 at. % excess of selenium and 500 mg of SeBr_4_. The ampule was melt sealed under high vacuum (<1 × 10^–3^ Pa using oil diffusion pump with LN_2_ trap) by oxygen–hydrogen torch. For the synthesis of Nb- and Re-doped WSe_2_ samples, the stoichiometry of starting materials corresponding to W_0.97_Nb_0.03_Se_2_ and W_0.97_Re_0.03_Se_2_ was used and keeping other procedures identical. The ampules were first placed in a horizontal muffle furnace and heated in several steps to avoid overpressure inside the ampule (500 °C for 50 h, on 600 °C on 50 h, and on 800 °C for 50 h). Heating rate of 1 °C/min was used and free cooling. The ampules were mechanically shaken to homogenize the materials between each heating step. For the crystal growth, the ampules were placed in a horizontal two-zone furnace. First, the growth zone was heated at 1050 °C while the source zone was kept at 800 °C. After 2 days, the temperature gradient was reversed, and for 7 days, the growth zone temperature was gradually decreased from 950 to 900 °C and the source zone temperature was increased from 1000 to 1050 °C. For the following 7 days, the thermal gradient was kept at 1050 °C for source zone and 900 °C for growth zone. Finally, the ampules were cooled to room temperature and opened in an argon-filled glovebox, and the resulting hexagonal crystals were collected. The CVT growth proceeded close to thermodynamic equilibrium condition, and the incorporation of dopant is limited by their solubility in MoSe_2_ and WSe_2_ at a given temperature. Concentration of dopant is dominantly influenced by the growth temperature, but also type of transport media (like various halogens) can have an influence on concentration of incorporated doping elements.

The crystals were exfoliated by a liquid-phase exfoliation procedure using high speed share force milling. In details, a quantity of 100 mg of bulk WSe_2_ (or doped WSe_2_) was introduced into a three-neck fused glass flask and subsequently immersed in a 100 mL solution consisting of a 50:50 mixture of deionized water and ethanol. Subsequently, the high-speed share force milling was performed for 1 h using an IKA T18 Ultra Turrax mixer at 20,000 rpm speed under an argon atmosphere. Exfoliated material was separated by filtration for further use.

### 2.3 Characterization

Powder X-ray diffraction (XRD) patterns of the samples were acquired using a Bruker D8 Advance, X-ray diffractometer (Cu Kα λ = 1.5406 Å, 40 kV, 40 mA, 2θ = 4° to 90°, scanning speed of 2° min^–1^). Scanning electron microscopy (SEM) images were recorded on a field emission gun electron source (FEG) of a SEM Tescan Lyra dual-beam microscope. Transmission electron microscopy (TEM) including high resolution TEM images were recorded on a Zeiss LIBRA 200 MC Cs scanning TEM tool. Elemental analysis using energy dispersive X-ray (EDX) analysis was also conducted using a detector of Oxford Instruments in the same tool. Raman spectroscopy (Renishaw InVia) was used to identify the characteristic modes of the synthesized materials. These measurements were performed at room temperature in the 100–1000 cm^–1^ spectral range using a He–Cd laser at an excitation wavelength of 532 nm. A 20× objective lens, along with a 5 mW power, was utilized to maintain optimal focus of the laser beam on the sample. The Brunauer–Emmett–Teller (BET) method was applied to assess the specific surface areas of the samples. Measurements were made using N_2_ adsorption–desorption with an instrument special surface area and NOVA Touch 4LX from Quantachrome Instruments. An inductively coupled plasma optical emission spectrometer (ICP-OES) with a radial view of the plasma (Arcos MV, Spectro) was used for Nb and Re determination. Before analysis, the samples were decomposed in a microwave digestion unit (Magnum II, Ertec, Poland) using a mixture of HNO_3_ and HF (both Analpure grade, Analytika s r.o., Czech Republic). Due to the complexity of spectral background for this matrix, the standard addition method was used for measurement. The digested samples were therefore diluted with deionized water and spiked with yttrium (serving as internal standard) and Nb/Re solutions (all Analytika s r.o., Czech Republic). Multiple wavelengths were observed for each element to identify possible spectral interferences. The determined concentrations of Nb and Re in WSe_2_ are approximately 998 and 4624 mg kg^–1^, respectively.

### 2.4 Electrochemical Measurements and Electrode Preparation

An Autolab PGSTAT 204 (Nova, Utrecht, The Netherlands) was used for all electrochemical measurements including cyclic voltammetry (CV), galvanostatic charge–discharge (GCD), chronoamperometry, and electrochemical impedance spectroscopy (EIS). EIS analysis was performed in the frequency range of 10 mHz to 100 kHz at zero voltage bias, under dark and light conditions. Chronoamperometry measurements were recorded using an LED source (λ = 420 nm) and a power of about 100 mW cm^–2^ (1 sun). The photo-ZIC was constructed using a zinc foil anode, a Whatman glass microfiber filter paper separator (18 mm diameter), and photocathode. Five wt % of carbon black powder, 5 wt % of PVDF, 2 mL of NMP, and 90 mg of WSe_2_ (or doped WSe_2_) were mixed and ultrasonicated for 60 min. The slurry, with a volume of 10 μL, was deposited using drop casting on a PET-coated ITO/Au substrate and utilized as the active material (Au nanoparticles were coated on ITO in order to facilitate the contact and stability of active materials on substrate (photocathode, see Scheme 1).

### 2.5 Density Functional Theory Calculations

We utilized VESTA to construct models for WSe_2_, Nb-WSe_2_, and Re-WSe_2_. Subsequently, we employed the density functional theory (DFT) simulation, specifically within Material Studio’s – CASTEP module, to investigate various properties including structure optimization, band structure, density of states (DOS), and optical properties (OP).

Our primary focus was to comprehensively examine and potentially enhance our understanding of the doping effect on WSe_2_ semiconductors. For the structural optimization, we employed ultrasoft pseudopotential to simulate the interaction between electron and ion cores, alongside geometric structure optimization and single point energy calculation, and the Perdew Burke Ernzerhof (PBE) in generalized gradient approximation (GGA) was used to describe the exchange-correlation function. A cutoff energy was set as 400 eV, and the K point was set to 4 × 2 × 2. The convergence threshold for SCF tolerance is 2 × 10^–6^ eV/atom between two electronic steps, and the maximum force on each atom is less than 0.01 eV/Å.

## 3 Results and Discussion

**Figure 1:**
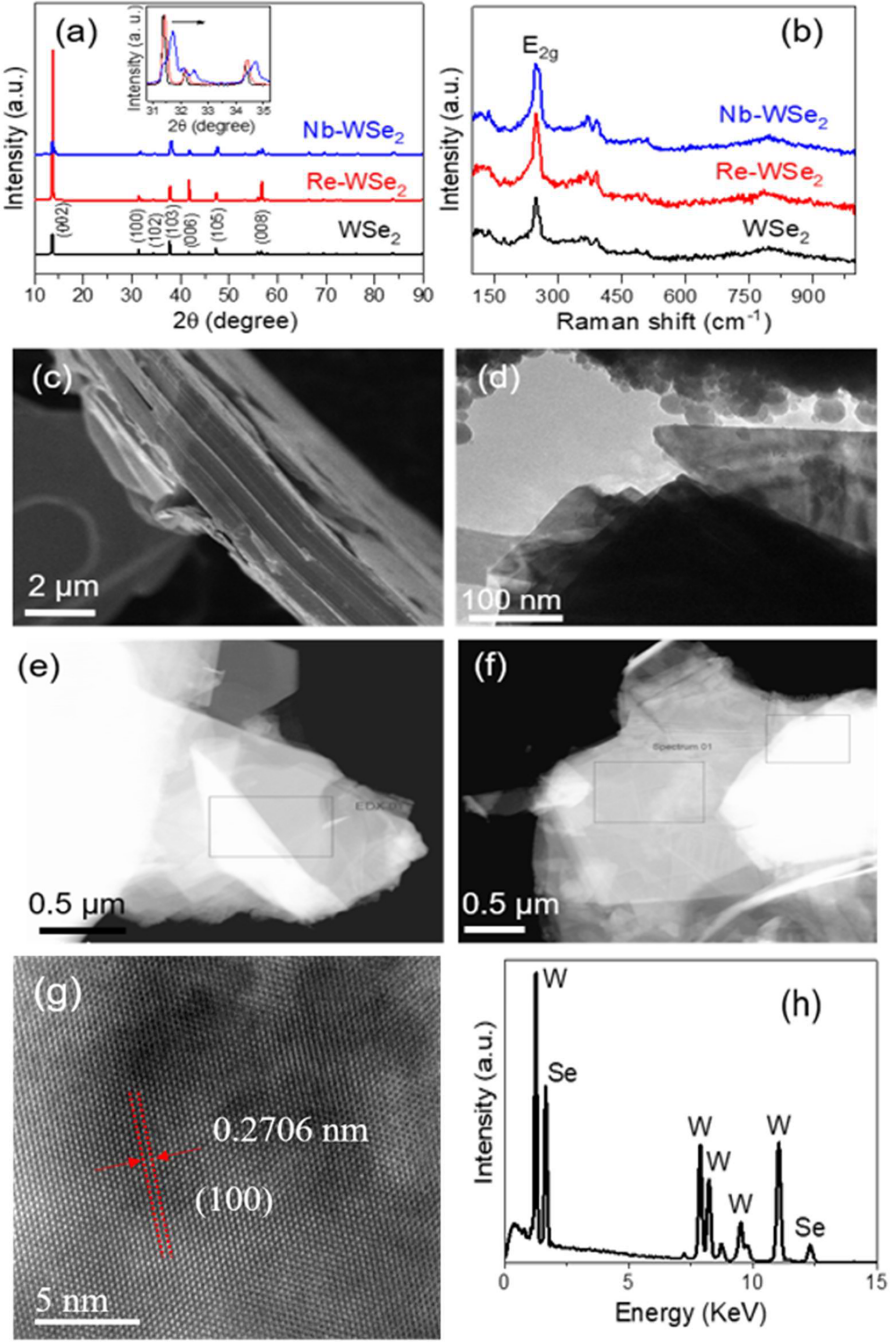
X-ray diffractograms of (**a**) pristine WSe_2_ (black) and Re-doped (red) and Nb-doped (blue) WSe_2_ after exfoliation. (**b**) Raman spectra of pristine WSe_2_ (black), Re-doped (red), and Nb-doped (blue) WSe_2_, excitation wavelength = 532 nm. Scanning electron microscopy (SEM) images of (**c**) bulk WSe_2_, STEM images of (**d**) exfoliated WSe_2_, (**e**) exfoliated Re-WSe_2_, and (**f**) exfoliated Nb-WSe_2_. (g) HRTEM of Re-WSe_2_ (image of the position in Figure S1). (h) EDX spectrum of the Re-WSe_2_ sample.

The diffractograms of the pristine, and Re- and Nb-doped WSe_2_ were recorded to analyze the crystallinity of the synthesized materials, as presented in Figure 1a. The XRD patterns of both undoped and doped samples exhibited a strong reflection peak at 2θ of 13.65°, ascribed to the (002) crystal plan of the hexagonal phase with a space group of *P*63/*mmc* (PDF 00-017-0887). The other peaks observed at around 31.47°, 34.42°, 37.87°, 41.74°, 47.41°, and 56.73° correspond to diffractions of (100), (102), (103), (006), (105), and (008) planes, respectively. After doping, no additional peaks were observed, which reflected the high purity of the crystallized phase and the successful incorporation of dopants into the backbone lattice. Preferential orientation in the (000l) direction was observed due to the van der Waals layered structure of WSe_2_.

Raman spectroscopy was used as a tool to gain further structural information about doped WSe_2_ samples (Figure 1b). The prominent peak located at 249.16 cm^–1^ was due to the well-known E_2g_ mode of hexagonal WSe_2_.^12^ The lower frequency modes at 370 and 390 cm^–1^ were not reported experimentally. Based on theoretical prediction, these modes could be assigned to out-of-plane and in-plane vibrations, respectively.^13^ No Raman peaks due to the presence of impurities were visible, indicating high phase purity. The mode displaying the highest intensity peak at 249.16 cm^–1^ represents the in-plane vibration of the W and Se atoms.^14,15^

The morphologies of pristine and doped WSe_2_ were analyzed by scanning electron microscopy (SEM), as shown in Figure 1**c-f**. It is clearly seen that the bulk WSe_2_ showed tightened layers, whereas, after liquid-phase exfoliation, the samples consisted of stacked nanosheets (Figure S1). It is obvious that the observed flakes have a reduced size with an area of about 20 μm^2^. Transmission electron microscopy (TEM) analysis was performed to study the existence of a flake of exfoliated WSe_2_ (Figure S2). The pristine and doped samples showed sheets and a large particle size. It seems that there is a mixture of bulk and exfoliated sheets. High-resolution transmission electron microscopy (HR-TEM) images of unaltered WSe_2_ and Re-doped WSe_2_ are displayed in Figures 1g and S1. The lattice interplanar spacing was determined to be approximately 0.2886, 0.2706, and 0.2879 nm for WSe_2_, Re-WSe_2_, and Nb-WSe_2_, respectively. This finding aligns with the (100) crystal plane of the hexagonal phase of WSe_2_.^16^ The HR-TEM and selected area electron diffraction (SAED) images (Figures S1 and S2) revealed the hexagonal arrangement of atoms, indicating the well-crystalline structure of the samples, which is consistent with the XRD diffractograms. Scanning transmission electron microscopy combined with energy dispersive X-ray spectroscopy (STEM-EDX) analysis of Re-doped WSe_2_ was carried out, and the detected elements are presented in Figure 1h. All peaks were overlapped with tungsten, which limits the quantitative analysis of Re in the sample. Considering that the spatial repartition of rhenium within the 3%/WSe_2_ stacked sheets is not homogeneous, the presence and location of rhenium in the whole sample are difficult to assess from HR-TEM and EDX analyses, which provide information at a very small scale. Unfortunately, due to the small difference between the atomic number of W and Re, it was difficult to detect Re in the WSe_2_ sample.^17^ Therefore, more quantitative analysis was conducted using inductively coupled plasma optical emission spectrometry (ICP-OES) combined with microwave-assisted sample digestion. The ICP-OES measurements confirmed the presence of Nb and Re doping elements in the WSe_2_ backbone. The concentrations of Nb and Re to WSe_2_ were approximately 998 and 4624 ppm, respectively.

The UV–visible spectroscopy was employed to investigate the optical absorption characteristics of the synthesized electrodes (Figure S3). The variation of absorbance with incident wavelength of exfoliated samples is similar to previous reports on WSe_2_,^18−20^ WS_2_, and MoS_2_.^21^ Re- and Nb-doped WSe_2_ electrodes exhibited superior absorption in the visible region, as compared to the undoped sample. This phenomenon could be due to the presence of defect states in the bandgap induced by the incorporation of dopants (Re, Nb). Multilayered and bulk WSe_2_ have been reported to have indirect band gap transition.^22^ Therefore, identification of the indirect band transition by UV–vis spectroscopy is a bit tricky, but the presence of a very large absorption band was observed. The band gaps of undoped and doped WSe_2_ were determined using the Tauc’s formula α*h*ν = *A*(*h*ν – *E*_g_)^n/2^, where *h*ν is the photon energy and *A* and *n* are constants. Figure S4a-f depicts the Tauc’s graphs using direct and indirect allowed transitions, in which the indirect band gap fits better the curves and the estimated band gaps were 1.91, 1.80, and 1.83 eV for WSe_2_, Re-WSe_2_, and Nb-WSe_2_, respectively. Although the fits may not provide precise values of band gap and band tails, their variation with doping remains a relevant and significant aspect, as shown in Figure S4.

Our theoretical calculations elucidated that the enhancement in the optical properties in Re-WSe_2_ primarily stems from the shift of the conduction band closer to the Fermi level, resulting in metallic behavior, as depicted in Figure S5. By analyzing the band structures displayed in Figure S6 a-c, we observed a decrease in the band gap of the WSe_2_ system from 1.44 to 0.366 eV upon the introduction of Nb atoms. This shift indicates electron transfer and enhances the material conductivity. To further understand the structural properties and evaluate the effect of Nb and Re doping, we calculated the density of states (DOS) and partial density of states (PDOS) of WSe_2_, and Nb- and Re-doped WSe_2_, as displayed in Figure S6 d-f. In Figure S6e,f, PDOS plots for doped Nb- and Re-WSe_2_ revealed the appearance of novel states near the Fermi level. These states emerge due to the presence of Nb and Re dopant atoms, altering the charge distribution within the system. It is evident that Nb and Re atom doping reduces the band gap of WSe_2_ and facilitates electron transfer between the valence and conduction bands, consistent with the experimental findings. The overlap of dopant atoms with W-5d orbitals confirms the chemical interaction between the dopant and W atoms, indicative of charge transfer mechanisms. Notably, compared to Nb-WSe_2_, Re-WSe_2_ exhibited a stronger d-orbital overlap, resulting in the shift of conduction band below the Fermi level and the creation of metallic behavior. This confirms the significant influence of Re doping on the electronic distribution of the WSe_2_ crystal, ultimately enhancing its conductivity.

Figure S7 displays the N_2_ adsorption–desorption isotherms of undoped WSe_2_, Re-WSe_2_, and Nb-WSe_2_, revealing the doping effect on the specific surface area of multilayered samples. The absence of the hysteresis loop typically suggests a nonmesoporous structure of the material. The specific surface areas of Re-WSe_2_ and Nb-WSe_2_ significantly increased, reaching 8.35 and 6.86 m^2^ g^–1^, respectively. This contrasts with the specific surface area of undoped WSe_2_ (2.22 m^2^ g^–1^), indicating a promoting effect of rhenium to store charge and increase the number of active sites.

## 5 Photoelectrochemical Performances of WSe_2_, Re-WSe_2_, and Nb-WSe_2_

The electrochemical performances, under dark and illuminated conditions, of the WSe_2_, Re-WSe_2_, and Nb-WSe_2_ photocathodes were evaluated against a zinc anode using CR2016 coin cells. The photocathodes were dispersed in NMP containing a slurry of 5% super P and 5% PVDF as conductive additive and binder, respectively. The CR2016 coin cells were manufactured as a proof of concept using a Zn foil as anode electrode in an aqueous ZnSO_4_ electrolyte. The selected voltage window range of 0.3 to 1 V in the binary system cell was meticulously fine-tuned to mitigate the dehydration of SO_4_^2–^ and Zn^2+^ ions and the undesired hydrogen and oxygen evolution reactions (Figure S8). The proposed photohybrid Zn-ion capacitor cell is composed of four components (WSe_2_ as the photocathode, separator, ZnSO_4_ as electrolyte, and zinc foil as anode). The main contribution of zinc anode is devoted to the storage mechanism, and we believe that there is no effect of zinc anode on the optical behavior of the cell. The photogenerated electrons in the undoped or Re (Nb)-doped WSe_2_-based photocathodes, under illumination, are transferred through PET-coated ITO/Au and the external circuit to reach the Zn anode. However, photogenerated holes engage in anion adsorption on the photocathode surface to raise the overall specific capacitance under illumination. This phenomenon occurs because of an energetically desirable pathway. As a result, a potential difference initiates between the photogenerated holes in the undoped or Re (Nb)-doped WSe_2_ based photocathodes and the transferred photoelectrons in the Zn anode, leading to the ionization of zinc sulfate.

**Figure 2:**
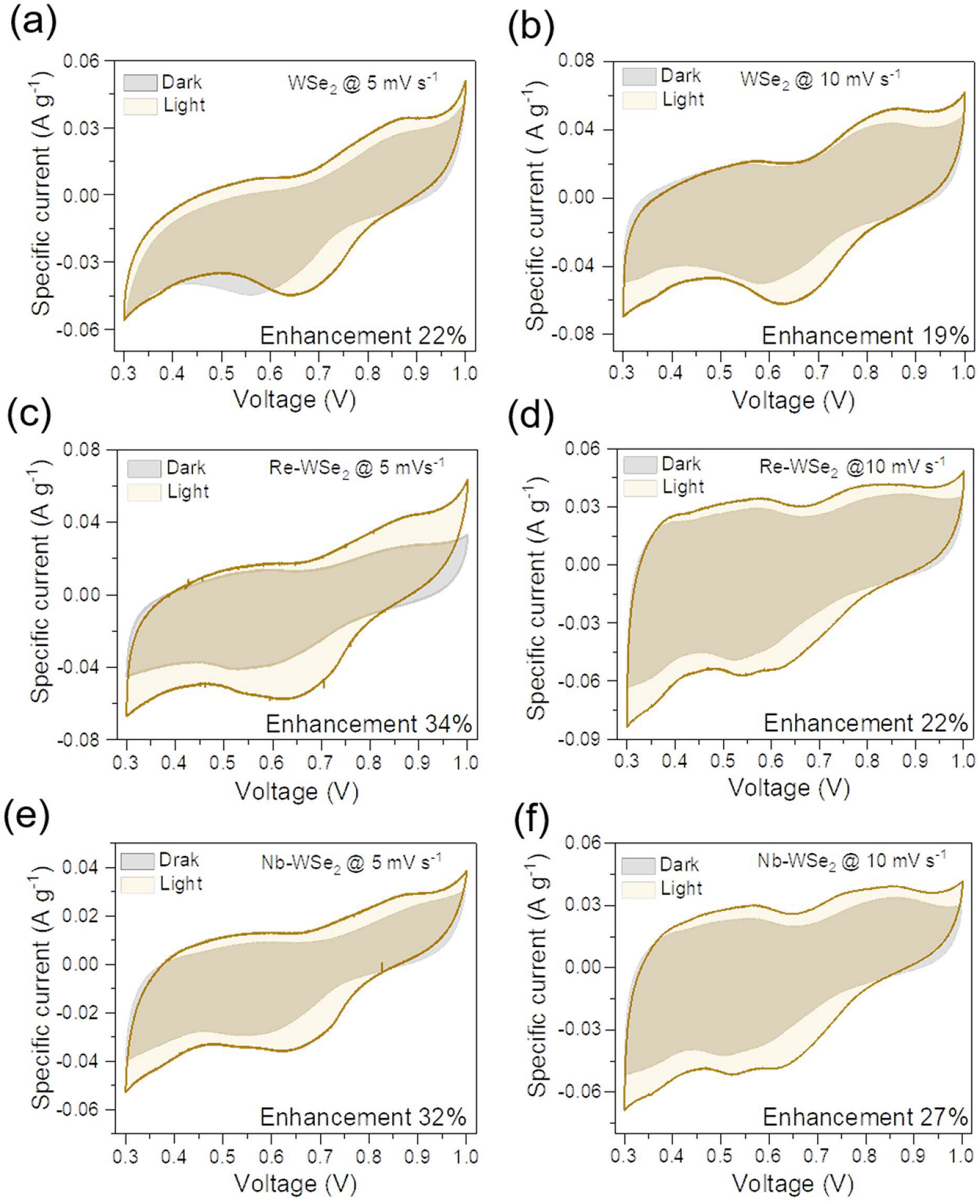
Comparative CV curves at 5 and 10 mV s^–1^ before and after irradiation for (**a**, **b**) pristine WSe_2_, (**c**, **d**) Re-doped WSe_2_, and (**e**, **f**) Nb-doped WSe_2_ samples.

Under illumination, the photogenerated electrons move to the WSe_2_ surface and accumulate on the backside of the zinc anode, interacting with Zn^2+^ from the electrolyte. On the photocathode, the photogenerated holes were transported to the anionic species within the electrolyte, initiating electric double layer capacitance (EDLC) and starting the photocharging procedure. The photoelectrochemical performances of all synthesized samples were assessed by cyclic voltammetry (CV), galvanostatic charge–discharge (GCD), and electrochemical impedance spectroscopy (EIS) measurements. CV curves in the dark and under illumination were performed on photo-ZICs WSe_2_, Re-WSe_2_, and Nb-WSe_2_, as depicted in Figure 2. All CV plots were carried out across a range of scan rates, from 5–300 mVs^–1^, within a potential window of 0.3 to 1 V. The capacitance enhancement can be determined through the following formula , where *C*_light_ and *C*_dark_ represent the specific capacitances in dark and irradiation conditions, respectively. The *C*_dark_ and *C*_light_ are the specific capacitance which were calculated using the following equation, , where *m* is the mass of active material, *k* is the scan rate, and *v*2 – *v*1 represents the potential window).^23^

It was found that exposure of the cell to visible light (420 nm, 100 mW cm^–2^) significantly improved the performance in charge storage of the photo-ZICs. Figure 2**a-f** shows the dependence of the capacity enhancement at different scan rates under dark and light conditions. The increased capacities of PCs under light could be related to the photocharging effect, wherein the photogenerated electron–hole pairs contributed to the improvement of charge storage process. The most significant capacity enhancement of about 34% was acquired at a low scan rate of 5 mV s^–1^ for the Re-doped WSe_2_. However, an improvement of 20–30% was recorded at higher scan rates. The broadness of the CV area implies an insertion of ionic charges into the multilayered structure of WSe_2_. We can infer that this CV shape can be attributed to the SO_4_^2–^ intercalation and redox reaction mechanism. As observed in Figure S9, a drastic reduction in the capacitance enhancement was evident when the scan rate increased above 100 mV s^–1^. The decrease at high scan rate could be attributed to the sluggish kinetics of electrochemical reactions, including ion diffusion, and the limited time for the formation of double capacitance.^24^

**Figure 3:**
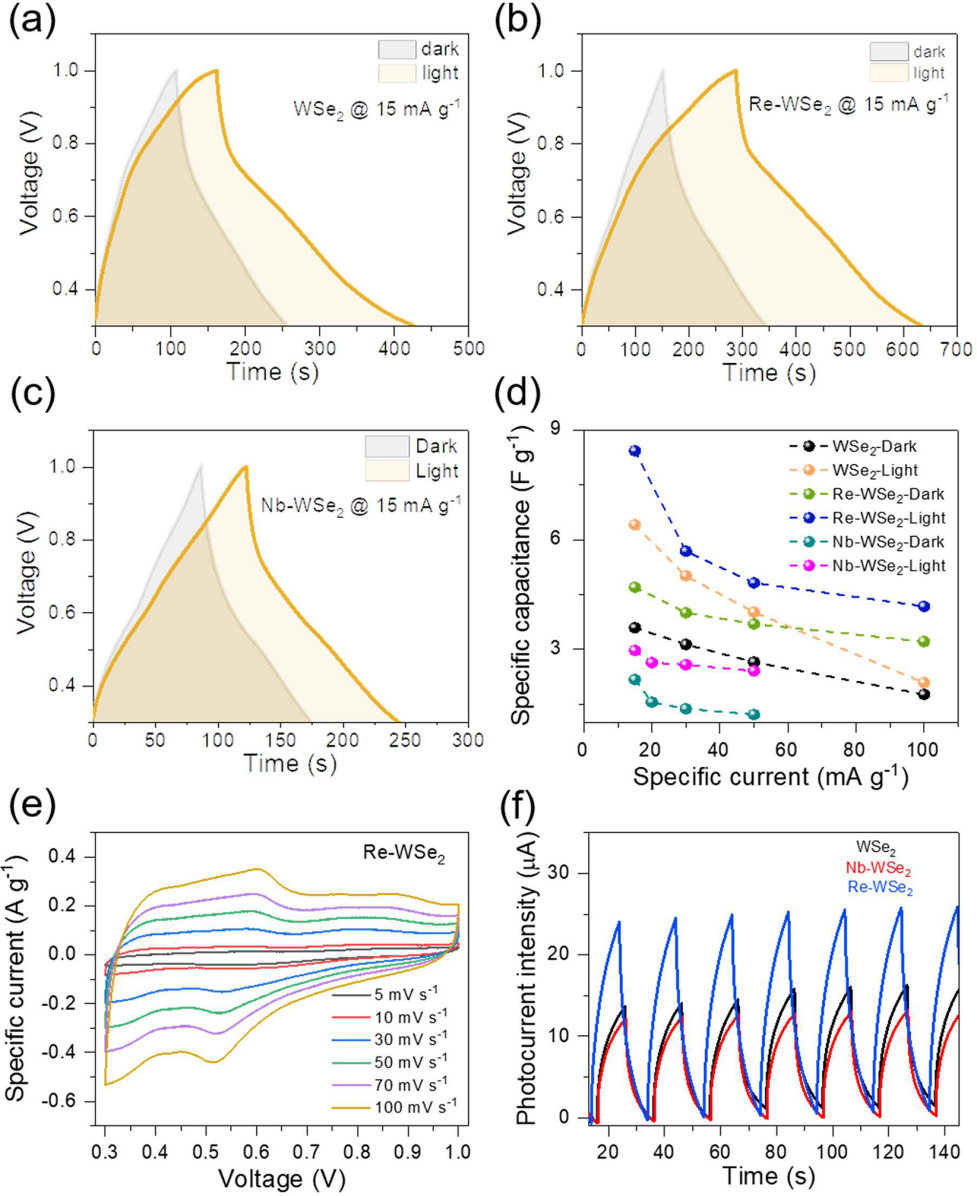
Comparative galvanostatic charge–discharge curves at a specific current of 15 mA g^–1^ in the dark and under irradiation of (a) WSe_2_, (b) Re-WSe_2_, and (c) Nb-WSe_2_. (d) Specific capacitance versus current plots of involved samples in the dark and under light. (e) Cyclic voltammograms at different scan rates. (f) Chopped light chronoamperometry measurements (10 s off, 10 s on at 0 V).

The photocathodes’ performances were additionally evaluated using galvanostatic charge–discharge tests (GCD) under dark and light conditions, as shown in Figure 3**a-c** and Figure S10a-g. First, we applied a galvanostatic discharge at a specific current of 0.4 A g^–1^. Next, we compared CD curves under photocharge and dark discharge at various specific currents. The GCD curves are symmetrical, suggesting the involvement of the electric double-layer capacitance behavior. The corresponding specific capacity enhancements at different specific currents are provided in Figure 3d. Effectively, capacity enhancement owing to irradiation reached 43% and 45% at a specific current of 15 mA g^–1^ for undoped and Re-WSe_2_, respectively. The specific capacitance, obtained from the GCD measurements under light of WSe_2_ and Re-WSe_2_, was about 6.41 and 8.43 F g^–1^, respectively. Moreover, the obtained specific capacitance values of Re-WSe_2_ at 30, 50, and 100 mA g^–1^ were 5.68, 4.82, and 4.17 F g^–1^, respectively. Meanwhile, WSe_2_ and Nb-WSe_2_ photoelectrodes exhibited lower specific capacitance values of about 4.01 and 2.41 F g^–1^ at 50 mA g^–1^, respectively. Nonlinear discharge curves of all investigated samples imply the pseudocapacitance nature of the synthesized electrodes.^25^ Next, the photo-ZIC device was discharged both in the dark and under visible light using various specific discharge currents. It was evident that light irradiation significantly influenced the charge and discharge processes. The photodischarge time was higher than dark-discharge time. When the cell was irradiated, there was photogeneration of electron–hole pairs, which means more effective charge transfer, which could delay the discharging time.

A discernible decrease in magnitude of the specific capacitance at high operating currents was noted. The superior capacity improvements under diverse applied currents could be assigned to the light interaction and the participation of photogenerated electron–hole pairs in the storage process. Figure 3e depicts the cyclic voltammograms of Re-WSe_2_ at different scan rates. Notably, at low scan rates, the collected curves did not feature a rectangular shape, evidencing the presence of both electric double-layer capacitor and pseudocapacitance mechanism.^26^ At higher scan rates, considerable positive and negative shifts were observed for the anodic and the cathodic peak, respectively. Moreover, the peak currents in the CV voltammograms exhibited a rise upon increasing the sweeping rate, demonstrating the rapid kinetics of electrochemical reaction around the active electrode and increased electrode polarization. Furthermore, at a high sweep rate, the CV curves maintained the same shape, inferring a good reversibility during fast charging/discharging processes. The photocurrent responses of the different photo-ZIC devices were acquired to assess the self-powered photosensibility by chronoamperometric measurements under shopped light illumination (420 nm LED source) at zero applied potential (bias). All investigated samples featured a photosensibility. The capacitor based on Re-WSe_2_ active electrode provided the highest photocurrent density value of almost 0.065 A g^–1^ (photocurrent of 26 μA), which is two times higher than that of undoped WSe_2_ (Figure 3f). This improvement in the photocurrent is a consequence of effective charge separation and transport. So, it could be unambiguously inferred that Re doping in the crystal lattice was responsible for this enhancement in photoactivity, considering that Re doping was reported as a promising method for light harvesting.^17^

**Figure 4:**
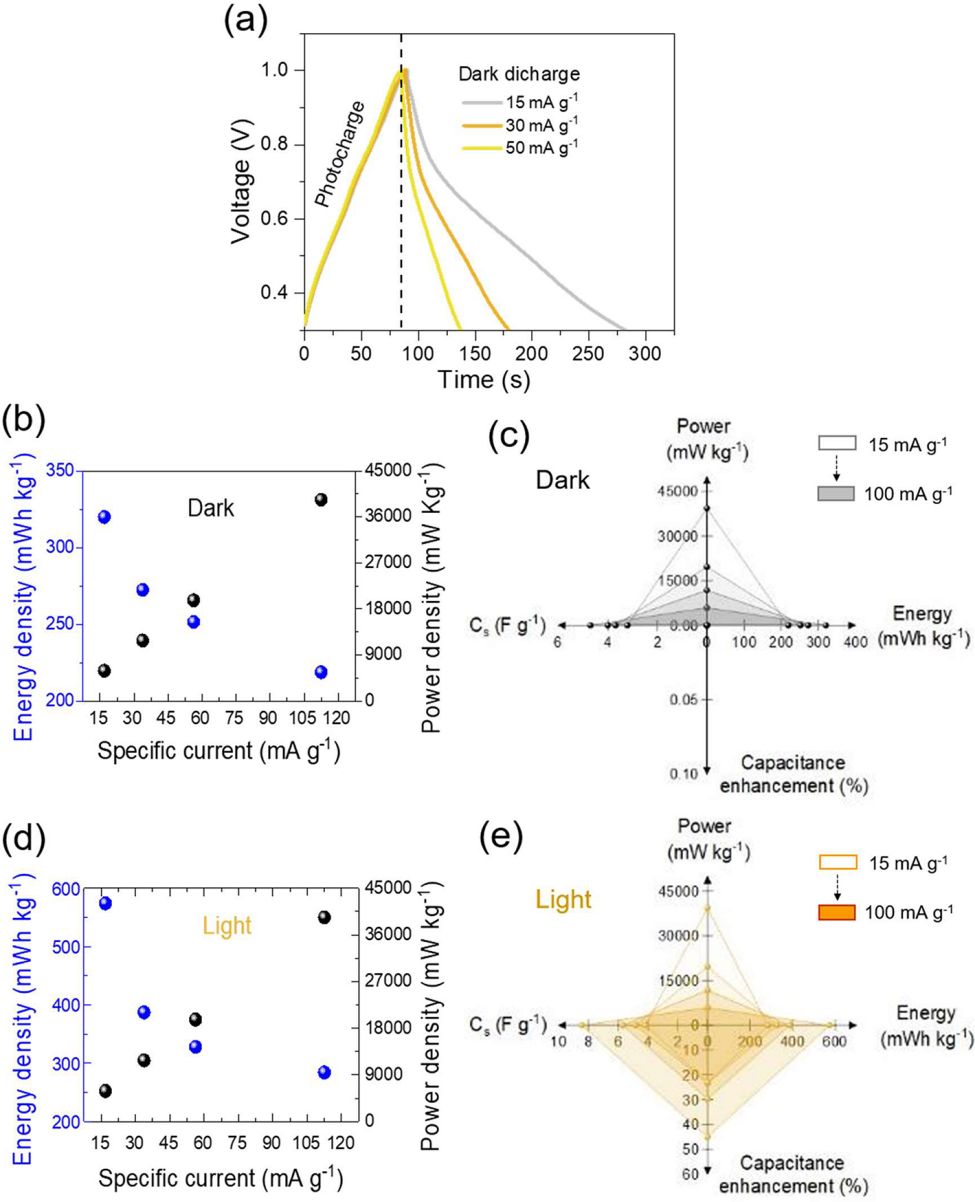
(a) Curves of photocharge (420 nm, 50 mW cm^–2^) and dark discharge at specific currents of 15, 30, and 50 mA g^–1^. (b, d) Energy and power density of Re-WSe_2_ before and after illumination supported by (**c**, **e**) spider charts.

Figure 4a displays photocharge of ZIC cell followed by galvanostatic discharge cycles under dark condition at specific currents of 15–50 mA g^–1^, which were used to calculate energy density and power density.^7^ Under irradiation, the Re-WSe_2_ electrode achieved an energy density of 574.2 mWh kg^–1^ at a power density of 5906 mW Kg^–1^ for a specific current of 15 mA g^–1^, while, under dark conditions, the maximum acquired energy density was approximately 319.9 mWh kg^–1^ under the same conditions (Figure 4**b**, **d**). Even at a higher power density of 39375 mW Kg^–1^, the sample acquired superior energy density, remaining at 285.0 mWh kg^–1^ (100 mA g^–1^) (spider chart, Figure 4**c**, **e**).

Other critical parameters for evaluating the capacitance performance of the photocathode are intrinsic resistance at the electrode–electrolyte interface using electrochemical impedance spectroscopy (EIS). A sinusoidal perturbation is a useful way to assess the electrical behavior of a sample, and the impedance is considered as a function of the frequency of the applied perturbation. The EIS measurements were carried out at a zero bias, in the dark, and under illumination (420 nm). The semicircle of the Nyquist plot at lower frequency usually indicates the occurrence of charge transfer at the semiconductor/electrolyte interface with a small radius reflecting an efficient charge transfer at the electrode–electrolyte interface. Furthermore, we observed the emergence of a second semicircle in the high-frequency region, a phenomenon that can be ascribed to charge transfer occurring at the semiconductor/substrate (Au-ITO) interface. Additionally, the presence of this second semicircle indicates the importance of considering the interface effects in investigating the electrical properties of semiconductors.

**Figure 5:**
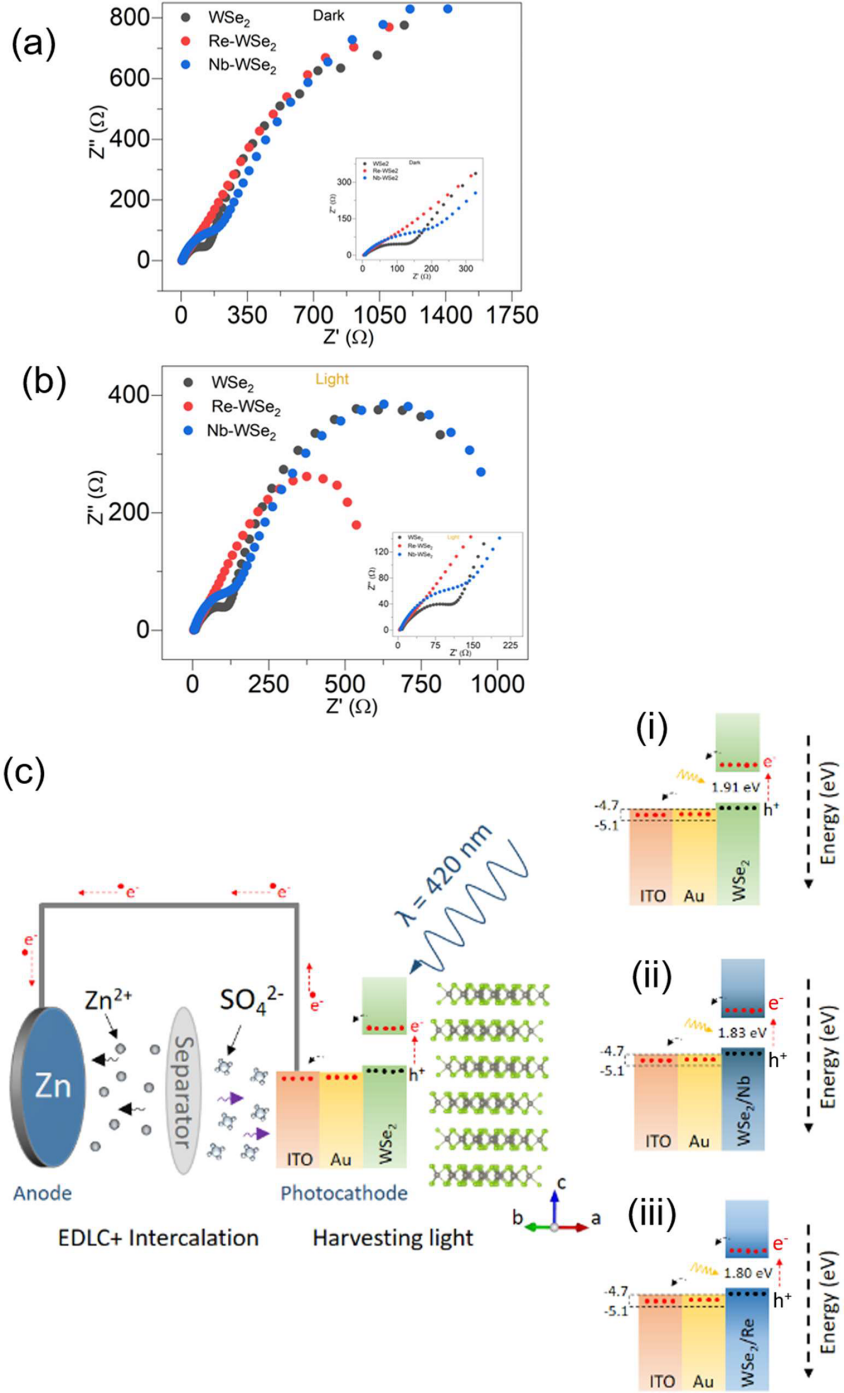
Nyquist plots of pristine WSe_2_, Re-doped WSe_2_, and Nb-doped WSe_2_ under (a) dark and (b) light. (c) Mechanism of photoenhanced electrochemical energy storage (photo-ZIC).

The Nyquist plots, obtained from the EIS measurements in the frequency range of 10 mHz to 100 kHz, are presented in Figure 5**a**, **b**. As expected, the doped samples under light irradiation showed a lower *R*_ct_, which supports the idea that incorporation of Re and Nb could enhance light absorbance and therefore facilitate transfer and separation of electron–hole pairs, which is beneficial in limiting the intrinsic resistance of less-conductive materials, consequently fostering a fast transport of charge from bulk to surface and to interface active electrode–electrolyte.^27^ The lowest radius of the semicircle was found for the Re-WSe_2_ sample, suggesting a better conductivity, which is consistent with the significant improvements of above photoelectrochemical properties. Similarly, in the high-frequency region, Re-WSe_2_ displayed the lowest radius compared to undoped and Nb-WSe_2_ electrodes. It is worth noting that Nb-WSe_2_ showed semicircles larger than those of pristine WSe_2_. We assume that this phenomenon can be attributed to the suboptimal interface between the Nb-WSe_2_ and the substrate, characterized by an energy level mismatch at their interface, leading to impedance in the charge transfer process, trapping efficient electron transport at the interface.

To understand the reaction kinetics, including ion diffusion and charge transport, fitted Nyquist impedance plots were obtained in the dark and illumination for pristine WSe_2_, Re-WSe_2_, and Nb-WSe_2_. Subsequently, equivalent circuits were provided, as shown in Figure S11 a-c and Table S1. Meticulous fitting of these spectra was performed, yielding remarkably low fitting errors for WSe_2_, Re-WSe_2_, and Nb-WSe_2_ with χ^2^ values of 0.142 (dark) and 0.144 (light), 0.105 (dark) and 0.082 (light), and 0.056 (dark) and 0.007 (light), respectively. According to the fitted results, each circuit consists of an electrolyte solution resistance in series with two parallel combinations of the charge transfer resistance (*R*_p_) and a constant phase element (CPE). It is seen that the undoped sample does not fit well with this diagram, and we replaced one CPE element by a capacitance to improve the fitting, as indicated in the equivalent circuits in the inset of Figure S11 a-c. It is evidenced that, under illumination, all samples exhibited lower resistance of electrolyte (*R*_s_) and charge transfer resistance (*R*_p_) of (*R*_s_ = 3.43 Ω; *R*_p_ = 151 (Ω)), (*R*_s_ = 2.29 Ω; *R*_p_ = 78 (Ω)), and (*R*_s_ = 4.81 Ω; *R*_p_ = 155 (Ω)) than in dark conditions with a resistance of (*R*_s_ = 5.46 Ω; *R*_p_ = 200 (Ω)), (*R*_s_ = 3. Ω; *R*_p_ = 90 (Ω)), and (*R*_s_ = 4.81 Ω; *R*_p_ = 222 (Ω)). The low resistance noted with the sample Re-WSe_2_ suggests that the photocathode material possesses significant electrical conductivity. This characteristic is advantageous for facilitating a rapid ion diffusion process. These results strongly support the superior rate capability of the Re-WSe_2_ electrode, especially under illumination conditions, where there is occurring an enhancement of photogenerated charge carrier separation, transport, and participation in the storage process.

We have shown that the presence of Re in WSe_2_ multilayers leads to an increase in the capacitance efficiency under light irradiation. In the following, we propose a description of the mechanism of photoconversion and storage in one single device (Figure 5**c**). Prior to that, it is essential to emphasize that our explanation is rooted on the knowledge that Re doping has the capability to create sub-band-states in the band gap, as already discussed in the UV–vis measurements. Under dark conditions, we observed that all samples provided non-negligible capacitance, which was around 4.7 F g^–1^ for Re-WSe_2_ due to the intercalation of ions from the electrolyte. When the cell was exposed to visible light, the initial pathway is the absorption of incident photon by sub-band state in the semiconductor, which allows the transfer and flow of photogenerated electrons to the zinc plate (anode side). In this case, the electrons could attract Zn^2+^ ions and form the first electric layer. Since Re doping introduces energy levels in the band gap, the holes created in the valence band interact with anionic charges (SO_4_^2–^). Incorporating Re or Nb ions into the WSe_2_, either by substituting W or Se atoms or occupying interstitial sites, is likely to promote the photogeneration of electron–hole pairs and induce defect states within the band gap. However, the photogeneration of electron–hole pairs requires the availability of electrons in the conduction band (or hole in the valence band), which can be challenging for materials with a large or indirect band gap; this may need further theoretical investigations to validate the existence of donor levels in the conduction band of WSe_2_. In the case of the Nb-WSe_2_ photoelectrode, visible light irradiation can potentially generate electrons in the conduction band and holes in the valence band, facilitated by the creation of narrowing sub-bands resulting from various defect states observed via UV–vis spectroscopy. Nonetheless, light absorption does not always translate to high photoconversion efficiency. Effective photogenerated charge separation is crucial for enhancing semiconductor performance. Additionally, under doping effect, the removal of atoms from the surface may lead to the trapping of electrons, a phenomenon that is potentially more adequate with Re doping. Considering these factors collectively, the suppression of the photogenerated electron–hole recombination increases the electron–hole lifetime, thereby improving the photoconversion performance.

In order to understand charge carrier density on doped samples, we further performed Hall measurements with the DX-100 Hall effect system. Cr/Au (5/35 nm) contacts were deposited on bulk samples via thermal evaporation in a van der Pauw geometry. The thickness of the bulk samples was 0.5 to 0.9 mm. Measurements were performed under a sweep of ±500 mT magnetic field at room temperature. For Nb-doped sample, bulk charge carrier density was 1.6 × 10^19^ cm^–3^ with p-type conduction that corresponds to active dopant concentration of 0.115 at%. On the other hand, Re-doped sample was n-type with comparatively lower bulk charge carrier density of 0.09 × 10^19^ cm^–3^, which corresponds to 0.048 at% active dopant. Hall mobility for both samples is depicted in Figure S12. Doping has led to an increase in the density of charge carriers, particularly electrons, which represents one of the advantages of this photocell device. Specifically, in an n-type semiconductor material, the doping process (*R*_e_) introduces additional free electrons, thereby enhancing the current density produced when light shines on the sample. This observation aligns with the findings from electrochemical impedance spectroscopy (EIS). Both electric double-layer capacitance and intercalation can be put forward to explain the enhancement of capacitance under visible light irradiation. The TMDC-based photo-ZICs stand out as indispensable energy storage devices, providing good electrochemical performance while remaining cost-effective, secure, and eco-friendly. Nonetheless, photo-ZIC is at a nascent stage, and enormous challenges remain that require mitigation, such as light absorption, interface contact, and stability. Our photocharged capacitance of up to 8.43 Fg^1–^ is better than those reported for some conventional solid-state supercapacitors including ternary oxide,^28^ 1T-WS_2_, which provided 2.3 mFg^–1^.^28^ However, doped WSe_2_ exhibits comparable^28^ and lower^9,29−31^ capability than other reported photo-ZIC cells, as summarized in Table S2. The enhancement in the photo-ZIC properties of WSe_2_ after Re doping can be ascribed to several factors, as indicated by the characterization results.^29^ Specifically, the incorporation of metals induced a reduction in the band gap and the creation of states within the band gap.^30^ This was corroborated by an increase in carrier density as confirmed by electrical measurements, along with a reduction in charge transfer resistance, which emerged as a significant influencing factor for photo-ZIC performance.^31^ Re-doped WSe_2_ demonstrated a notable increase in photodriven capacitance, particularly evident from the EIS measurements, which revealed the lowest reduced charge transfer resistance across the interface. Moreover, the optical behavior of the Re-doped WSe_2_ sample suggests its enhanced ability to absorb more incident light compared to that of both undoped and Nb-doped WSe_2_ electrodes. This aspect is crucial in improving the light harvesting capacity of materials.

**Figure 6:**
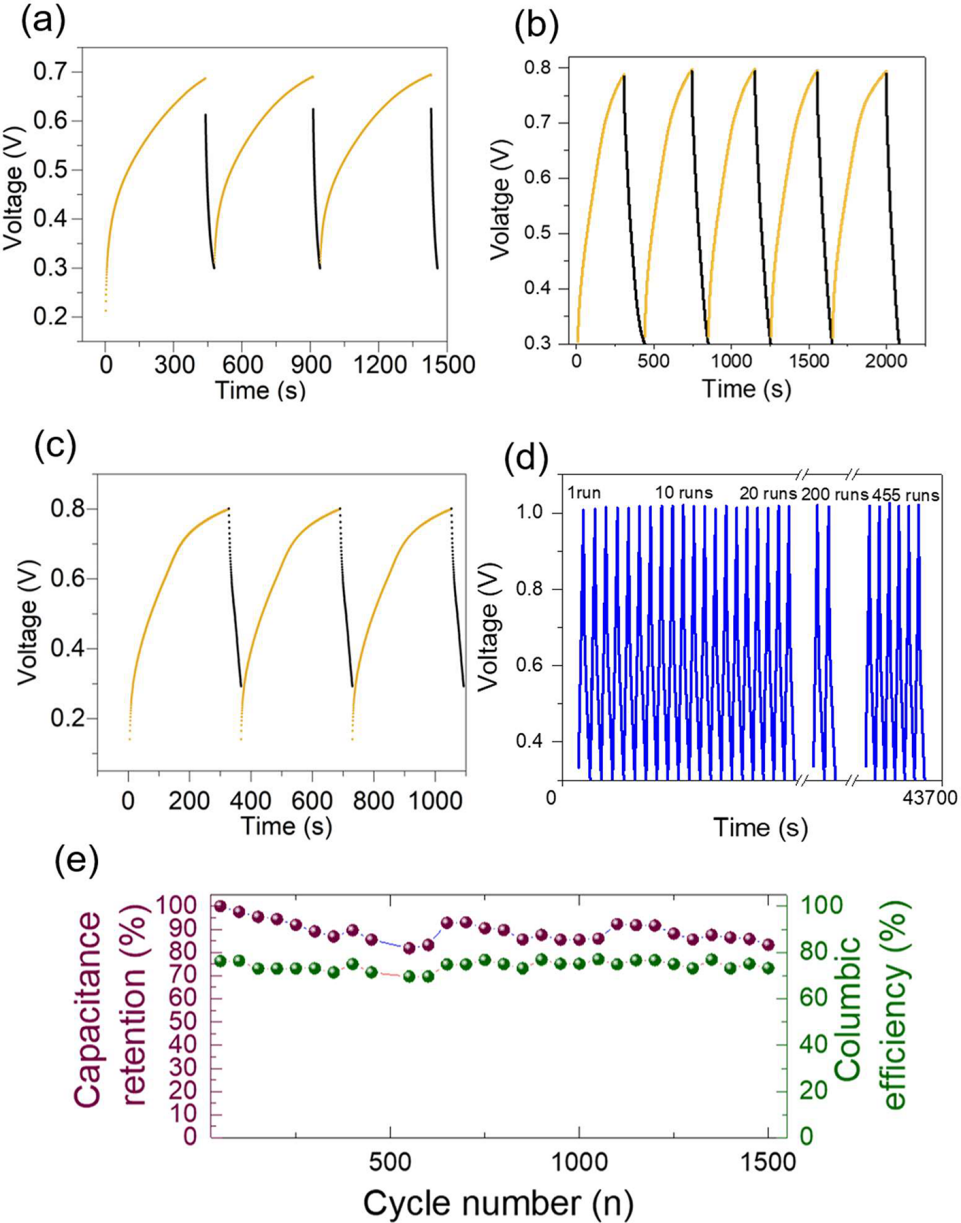
Photocharging/dark discharge at 0 V of (**a**) pristine WSe2, (**b**) Re-WSe_2_, (**c**) Nb-WSe_2_, (**d**) cyclic stability of Re-WSe_2_ of 500 cycles, (**e**) capacitance retention and Coulombic efficiency of Re-WSe_2_.

Furthermore, the stability of the assembled cell was studied by repeated the voltage floating (the cell was charged only by light at 0 V as external potential) followed by dark discharge (Figure 6**a-c**). Effectively, the ZIC-based Re-WSe_2_ photocathode at zero bias reached a voltage response of 0.8 V in lower time (almost 300 s). Under the same conditions, pristine WSe_2_ and Nb-WSe_2_ attained 0.7 V (within 440 s) and 0.8 V (within 330 s), respectively. It confirms that light is responsible for this enhancement in photocharging and storage through photogeneration of electron–hole pairs. After five runs, the specific capacitance remained unchanged, indicating the stability of the material. To assess the enduring stability of the photo-ZIC, 1500 cycles of GCD under dark conditions were carried out at a specific current of 50 mA g^–1^, revealing a low capacitance retention of almost 20% over 1500 runs of the photo-ZIC, as illustrated in Figure 6**c**. However, pristine WSe_2_ showed a low capacitance retention of about 25% after 500 cycles, as shown in Figure S13. After stability, the SEM images (Figure S14) of Re-WSe_2_ revealed a similar morphology before stability assessment (stacked sheets); however, the conductivity recognized some decrease due to some Zn precipitation or dendrite formation, which could explain the loss of capacitance after excessive cycles.

## 4 Conclusion

In this work, we synthesized multilayered WSe_2_ and Re- and Nb-doped WSe_2_ photocathodes *via* chemical vapor transport, followed by a liquid-phase exfoliation method. The photoelectrode, consisting of doped WSe_2_ dropped on PET-coated ITO/Au substrate, was paired with a Zn foil, serving as an anode, to create a single device known as photo-ZIC cell. This innovative cell demonstrates multifunctional capabilities, which start with efficient harvesting of visible light followed by charge storage. Compared to the pristine sample, the Re-doped WSe_2_ showed better visible light absorption. Therefore, the Re-WSe_2_ exhibited a superior photocurrent value in accordance with the good performance in capacitance of photo-ZIC cell due the efficient light harvesting and storage. A capacitance enhancement of up to 45% under light was observed in that photo-ZIC. Under similar conditions, the pristine WSe_2_ and Nb-doped WSe_2_ recorded photoenhanced induced capacitance of 43% and 27% at 15 mA g^–1^ and supplied an energy density of 436.4 mWh kg^1–^ and 202 mWh kg^1–^, respectively. Also, the Re-doped WSe_2_-based device delivered a specific energy density of 574.2 mWh kg^1–^ at a power density of 5906 mW kg^1–^ and at 15 mA g^–1^. The work opens the gate to more development of new photoelectrochemical energy storage devices based on TMDCs including ZICs and ZIBs.

## Data Availability

The datasets generated during and/or analyzed during the study are accessible via the Zenodo repository: 10.5281/zenodo.11543483.

## Supporting Information Available

Experimental details, characterization methods including SEM images; HRTEM images; selected area electron (SAED) diffraction patterns; UV–visible measurements and extrapolated band gap; BET measurements; cyclic voltammetry curve of Re-doped WSe_2_ based photocathode in the voltage range from 0 to 1.2 V; CV curves at 100 mV s^–1^ in the dark and under illumination; Comparative galvanostatic charge–discharge curves at specific currents of 30, 50, and 100 mA g^–1^ in the dark and under irradiation; Fitting of Nyquist plot and equivalent circuit diagram; Hall measurements of Re-WSe_2_ and Nb-WSe_2_; cyclic stability of pristine WSe_2_; SEM images of the active photocathode before and after stability test (PDF)
