# Supplementary material for: 2D Rhenium- and Niobium-Doped WSe2 Photoactive Cathodes in Photo-Enhanced Hybrid Zn-Ion Capacitors
Source: ACS Appl Nano Mater. 2024 Jun 18;7(12):14102–14. doi: 10.1021/acsanm.4c01405 (PMC11220785; doi:10.1021/acsanm.4c01405)
Supplement: Supplementary file 1 — an4c01405_si_001.pdf [file an4c01405_si_001.pdf]

Supporting Information

2D Rhenium- and Niobium-Doped WSe_2 Photoactive Cathodes in Photo-Enhanced Hybrid Zn-Ion Capacitors

Monaam Benali[1], Jalal Azadmanjiri[1], Martin Loula[2], Zhongquan Liao[3], Rui Gusmão[1], Amutha Subramani[1], Kalyan Jyoti Sarkar[1], Rabah Boukherroub[4], Zdeněk Sofer[1*]

[1] Department of Inorganic Chemistry, University of Chemical and Technology Prague, Technicka 5, 166 28 Prague 6, Czech Republic

[2] Institute of Organic Chemistry and Biochemistry of the Czech Academy of Sciences, Flemingovo nám. 2, 16610, Prague 6, Czech Republic

[3] Fraunhofer Institute for Ceramic Technologies and Systems IKTS, Maria-Reiche-Straße 2, 01109 Dresden, Germany

[4] Univ. Lille, CNRS, Univ. Polytechnique Hauts-de-France, UMR 8520, IEMN, F-59000 Lille, France

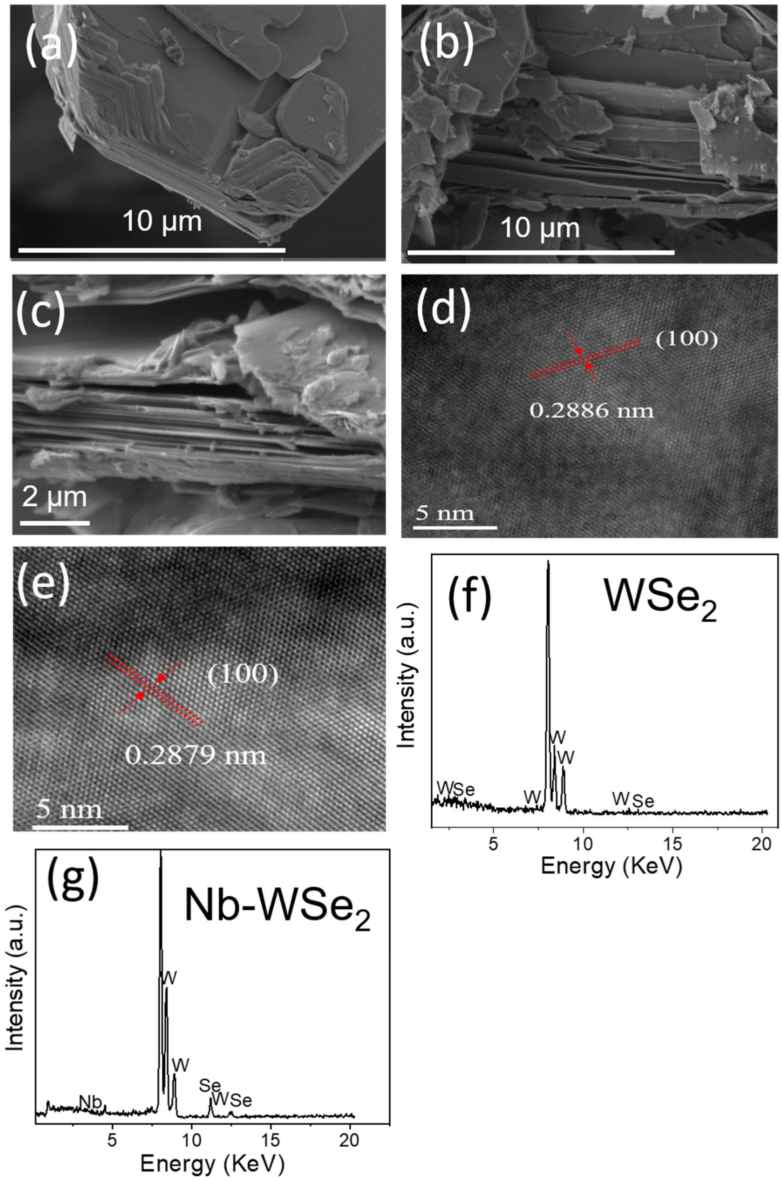
(a)
10 μm
(b)
10 μm
(c)
2 μm
(d)
(100)
0.2886 nm
5 nm
(e)
(100)
0.2879 nm
5 nm
(f)
WSe2
Intensity (a.u.)
Energy (KeV)
(g)
Nb-WSe2
Intensity (a.u.)
Energy (KeV)

Figure S1. SEM images of exfoliated (**a**) WSe_2, (**b**) Re- WSe_2, (**c**) Nb- WSe_2, (**d**) HRTEM of WSe_2, (**e**) HRTEM of Nb- WSe_2 (image of position in Figure S2). **(f)** and (**g**) EDX spectra of the WSe_2 and Nb-WSe_2 samples, respectively.

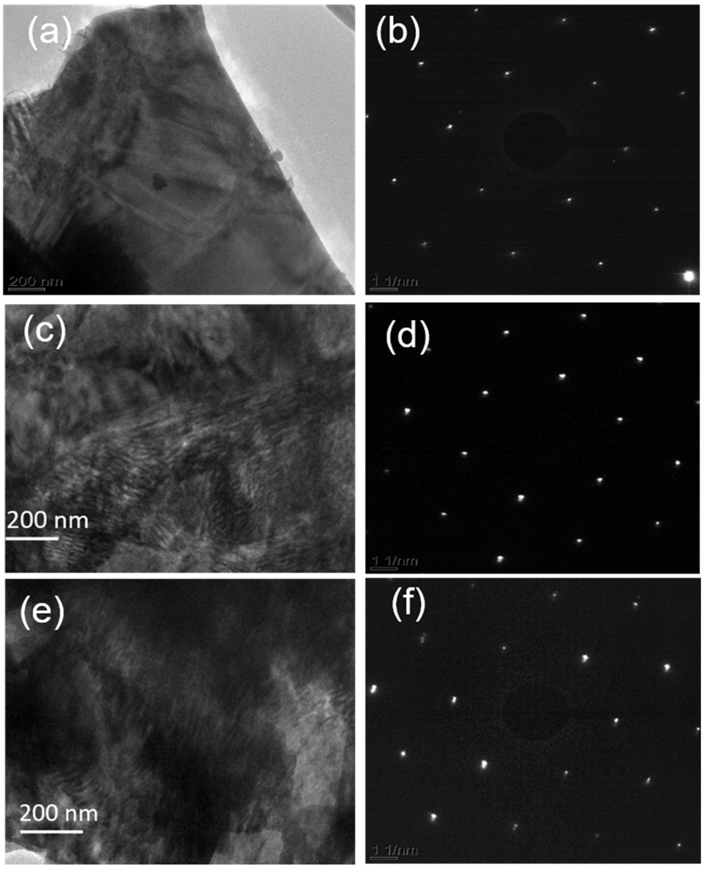

Figure S2. TEM images with the corresponding selected area electron (SAED) diffraction patterns of pristine WSe_2 (**a**, **b**), Re- WSe_2 (**c**, **d**), and Nb- WSe_2 (**e**, **f**).

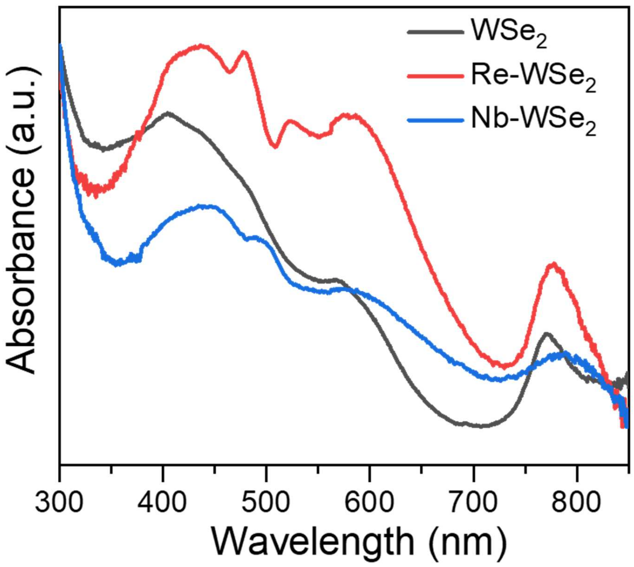

Figure S3. UV-visible spectra of pristine WSe_2 (black), Re- WSe_2 (red), and Nb-WSe_2 (blue).

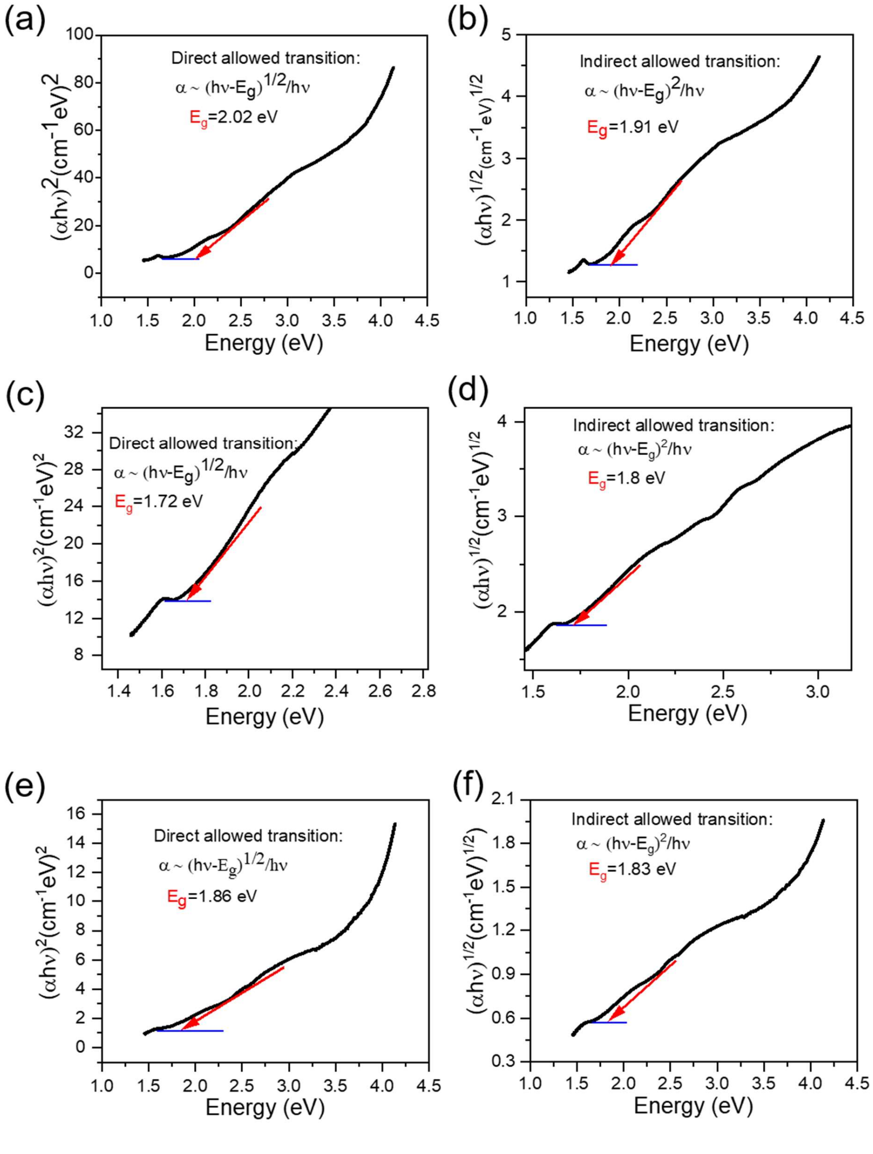

Figure S4. Tauc plots of pristine WSe_2 (**a** and **b**), Re- WSe_2 (**c** and **d**), and Nb- WSe_2 (**e** and **f**).

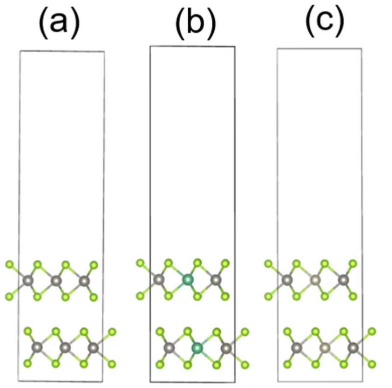

Figure S5. Optimized structures of **(a)** WSe_2, **(b)** Nb-WSe_2 and **(c)** Re-WSe_2.

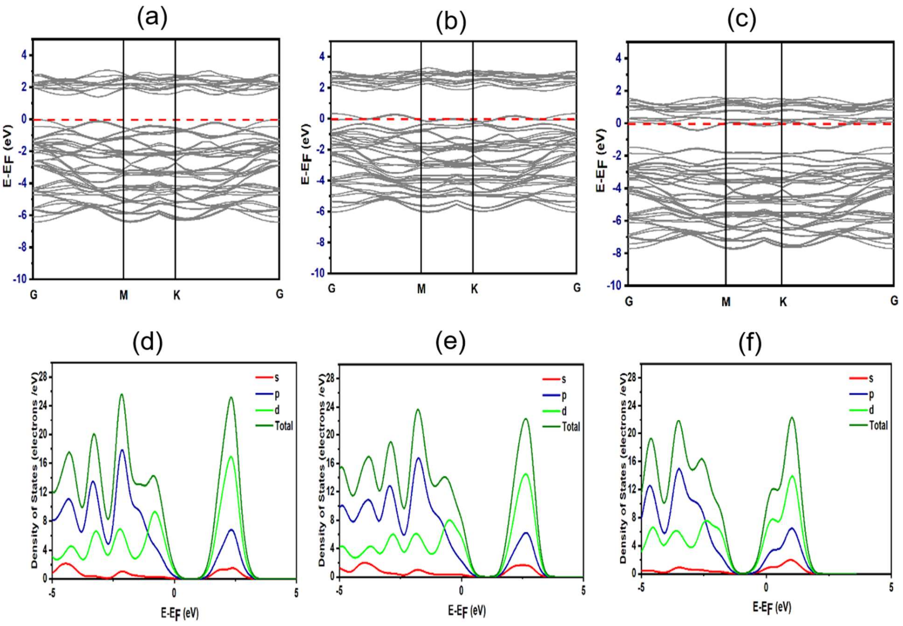

Figure S6. Electronic band structure of (**a**) WSe_2, (**b**) Nb-WSe_2 and (**c**) Re-WSe_2. Partial and total density of states for (**d**) WSe_2, (**e**) Nb-WSe_2 and (**f**) Re-WSe_2.

Okomentoval(a): [r1]: better to add what s, p, and d stand for in d,e,f

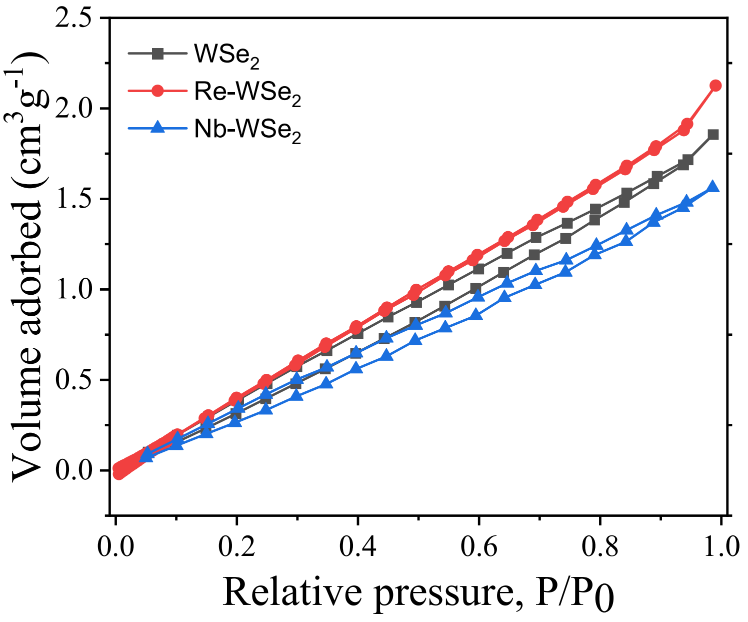

Figure S7. BET measurements of pristine WSe_2 (black), Re- WSe_2 (red), and Nb-WSe_2 (blue) samples.

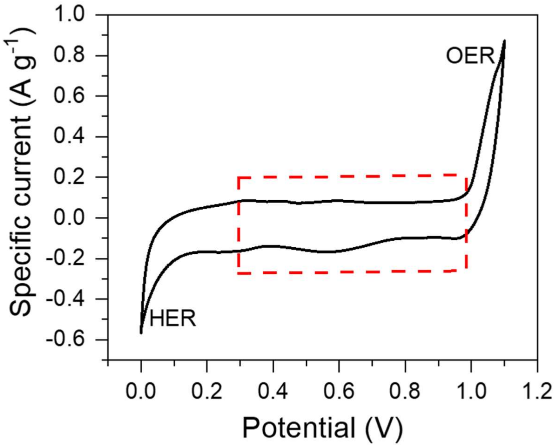

Figure S8. CV of WSe_2 in the potential range of 0 to 1.1 V, scan rate = 5 mVs^{-1}.

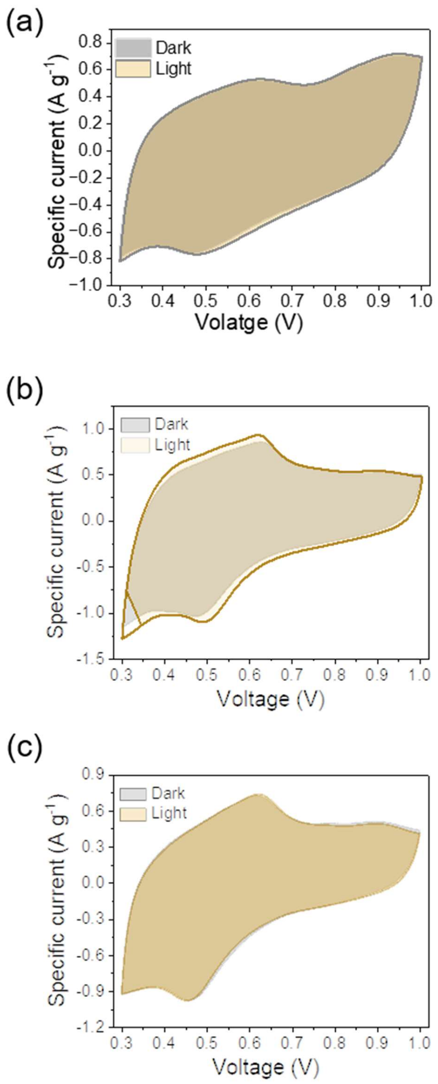

Figure S9. Comparison of CV curves at 100 mV s^{-1} in the dark and under illumination for (**a**) undoped WSe_2, (**b**) Re- WSe_2 and (**c**) Nb- WSe_2 samples.

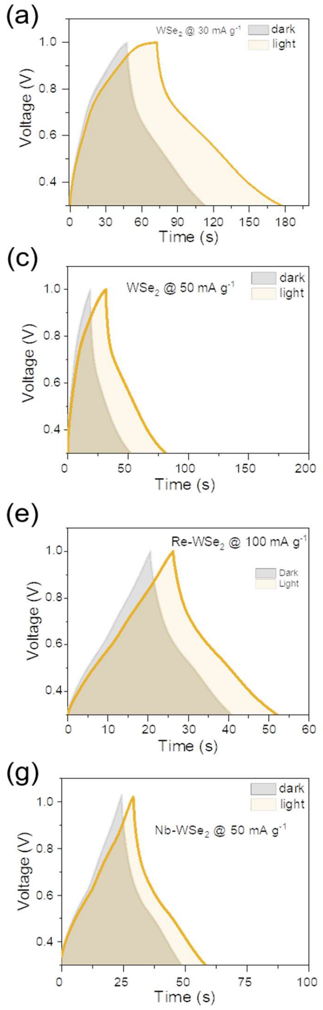

Figure S10. Comparative galvanostatic charge-discharge curves at specific currents of 30, 50 and 100 mA g^{-1} in the dark and under irradiation of **(a, c, f)** WSe_2, **(b, d, e)** Re-WSe_2 and **(g)** Nb-WSe_2.

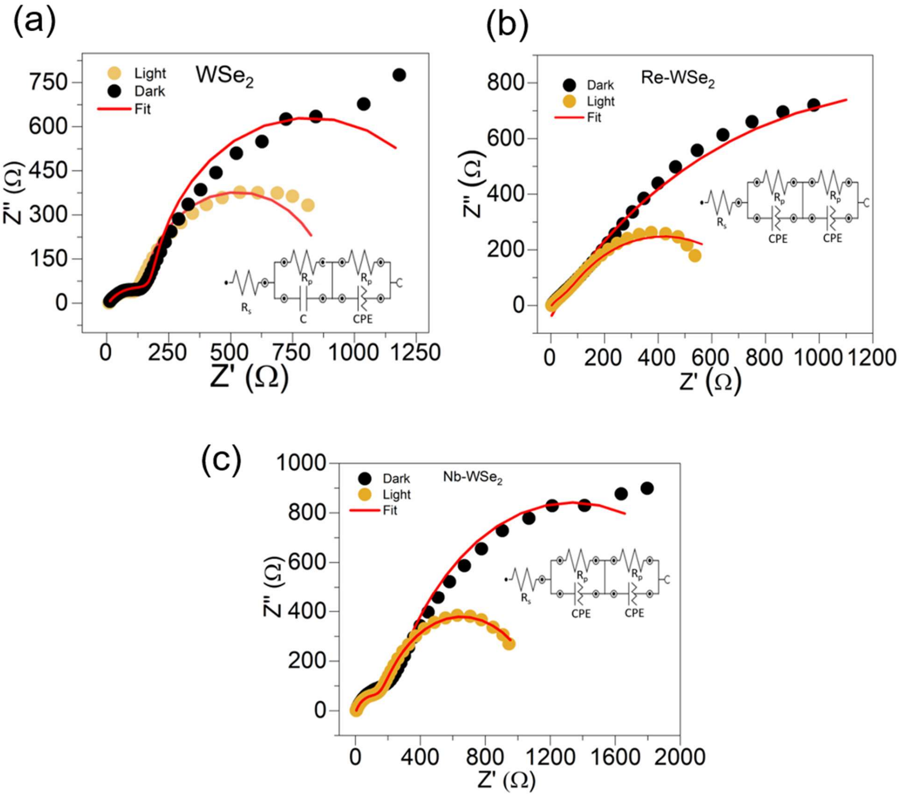

Figure S11. Fitting of Nyquist plots and equivalent circuit diagrams (insets) of **(a)** pristine WSe_2, **(b)** Re-WSe_2, and **(c)** Nb- WSe_2, under dark and light irradiation (420 nm, 100 mW/cm^2).

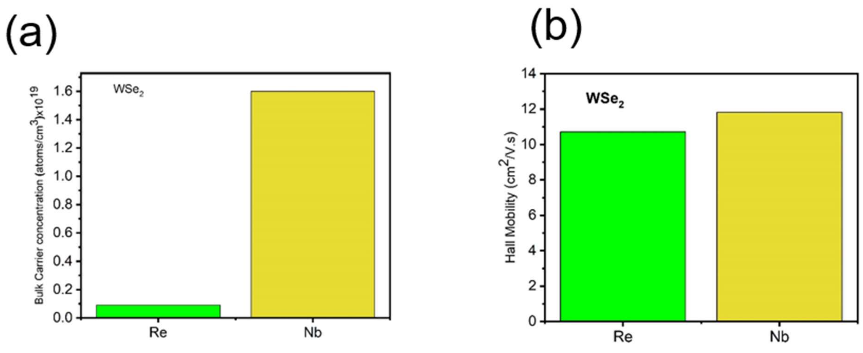

Figure S12. Hall measurements of Re-WSe_2 (green) and Nb-WSe_2 (yellow): (**a**) Charge carrier concentration, (**b**) Hall mobility.

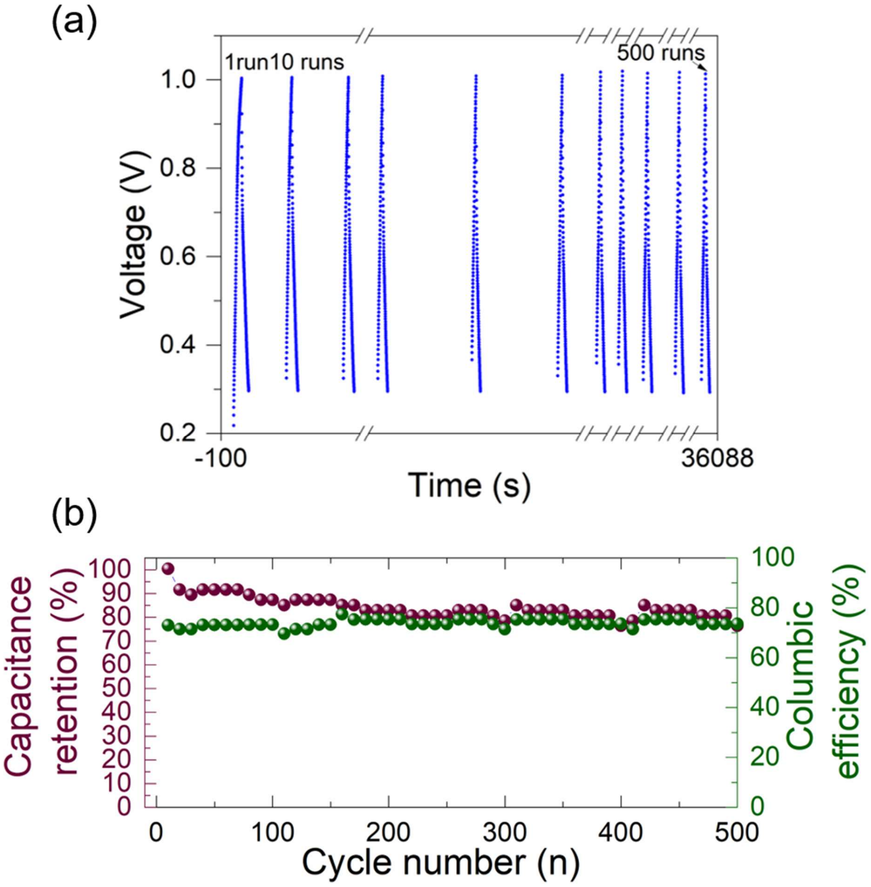

Figure S13. (a) Cyclic stability for 500 cycles, and **(b)** capacitance retention and coulombic efficiency of pristine WSe_2.

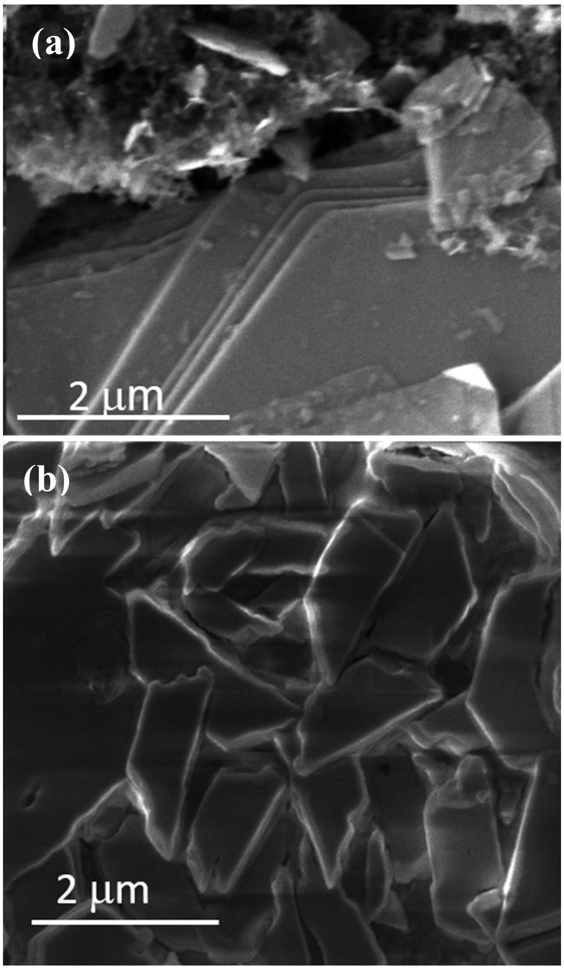

Figure S14. SEM images of the active photocathode Re-WSe_2 before **(a)**, and after **(b)** stability test.

Table S1. Comparison of parameters obtained from equivalent circuit in dark and under light irradiation for WSe_2, Re-WSe_2, and Nb-WSe_2.

Dark				Light	
**Sample**	**Elements and Parameters**	**Value**	**Estimated Error (%)**	**Value**	**Estimated Error (%)**
WSe_2	R_s	5.4609 (Ω)	13.983	7.0042 (Ω)	3.436
	R_p	1248.7 (Ω)	3.607	746 (Ω)	2.638
	C	692 (μF)	2.764	729 (μF)	2.638
	R_p	200 (Ω)	3.631	151.32 (Ω)	2.600
	Y_0	217 (μMho^s^N)	11.183	0.00017787 (μMho^s^N)	8.751
	N	0.55438	2.658	0.58309	1.732
	χ^2	0.14277		0.14472	
Re-WSe_2	R_s	2.8311 (Ω)	2.265	2.9125 (Ω)	2.299
	R_p	2429.9 (Ω)	13.198	698.12 (Ω)	18.939
	CPE (Y0)	853 (μMho^s^N)	3.079	1.17 (mMho^s^N)	8.000
	CPE (N)	0.71493	4.595	0.77378	1.832
	R_p	90.28 (Ω)	21.478	78 (Ω)	6.729
	CPE (Y0)	680 (μMho^s^N)	8.751	1.15 (mMho^s^N)	3.302
	CPE (N)	0.611	9.032	0.55111	4.023
	χ^2	0.10593		0.082566	
Nb-WSe_2	R_s	4.8138 (Ω)	1.554	4.8482 (Ω)	0.582
	R_p	2233 (Ω)	3.845	1000.1 (Ω)	1.219
	CPE (Y0)	0.00045154	2.273	0.00011145	1.887
	CPE (N)	0.81969	1.972	0.68307	0.330
	R_p	222.79 (Ω)	4.357	155.05(Ω)	1.289
	CPE (Y0)	9.9315E-05	4.469	0.00060727	0.958
	CPE (N)	0.69223	0.797	0.82033	0.740
	χ^2	0.05653		0.0073664	

Table S2. Comparison of different photo-enhanced electrochemical energy storage systems.

Photo-electrochemical energy storage system	Light-wavelength	Electrolyte	Voltage response	Specific capacitance	Energy and Power-densities	Ref.
V doped ternary Zn-Ni-Co oxide nanostructure	-	KOH/PVA	-	2960 mFg^{-1} @ $0.02Ag^{-1}$	148 mW h kg^{-1}, 12000 $mWKg^{-1}$ @$0.02Ag^{-1}$	28
1T-WSe_2	-	1M H_2SO_4	-	2813 μFcm^{-2}	-	29
CdS/ZnO	455 nm	3M $Zn(CF_3SO_3)_2$ aqueous electrolyte	800 mV	50 $mAhg^{-1}$	30 $WhKg^{-1}$, 100 WKg^{-1}	9
Sulfur-, tungsten-doped TiO_2 nanotube supercapacitor	λ = 533 nm, intensity ~ 100 mW cm^{-2}	0.5 M H_2SO_4	400 mV	31 mFg^{-1} @ 0.23 $mAcm^{-2}$	6.21 Wh cm^{-2} And 399 W cm^{-2} @ 0.23 mA cm^{-2}	30
2D g-C_3N_4	λ = 420 nm, intensity ~ 50 mW cm^{-2}	2 M $ZnSO_4$	850 mV	11377 mFg^{-1}	~ 668 mWh kg^{-1}, 1625 mW kg^{-1} @ 0.005 A g^{-1}	7
Germanane-cyanoethyl (Ge–C_2–CN)	λ = 435 nm, intensity ~ 50 mW cm^{-2}	2 M $ZnSO_4$	1000 mV	6 Fg^{-1}	550 mWh Kg^{-1}, 31000 mW Kg^{-1} @ 0.060 A g^{-1}	31

Niobium Carbide MXene	λ = 435 nm, intensity ~ 50 mW cm^{-2}	2 M $ZnSO_4$	1000 mV	27 Fg^{-1}	2.4 Wh Kg^{-1}, 40 WKg^{-1} @ 0.030 A g^{-1}	22
Re-WSe_2	λ = 420 nm, intensity ~ 100 mW cm^{-2}	2 M $ZnSO_4$	800 mV	8.43 Fg^{-1}	574.21 mWh Kg^{-1}, 5906 mW Kg^{-1} @ 0.015 A g^{-1}.	This work
